# Integrating Predictive Analytics into Hospital Automation: Comparative Evaluation of Prediction Models for Patient Monitor Demand in Operating Rooms

**DOI:** 10.3390/bioengineering13091073

**Published:** 2026-09-15

**Authors:** Shaohua Yin, Sujuan Yu, Zhenlin Liu, Boqi Jia, Chenxi Shi, Yanfang Xu, Yun Tian, Xiaoxiao Luan

**Affiliations:** 1 Medical Engineering Department, Peking University Third Hospital, Beijing 100191, China; ysh@bjmu.edu.cn (S.Y.); bysy_ysj@126.com (S.Y.); zhenlin008@163.com (Z.L.); 1963192507@bjmu.edu.cn (B.J.); 18511284628@163.com (C.S.); xyf_bsygc@126.com (Y.X.); 2 Orthopedics Department, Peking University Third Hospital, Beijing 100191, China

**Keywords:** machine learning model, patient monitor, health resource allocation, prediction, equipment service age

## Abstract

Predictive analytics has emerged as an important component of hospital automation by enabling proactive resource management and data-driven decision-making. Efficient allocation of patient monitors in operating rooms represents a practical application where forecasting can support perioperative safety and resource management. This study compared five forecasting models for predicting patient monitor availability using integrated clinical, operational, and equipment management data to identify appropriate forecasting approaches for operating room resource planning. We conducted a retrospective longitudinal study using monthly surgical operational data from the anesthesia information system and equipment-related data from the medical equipment management system of a tertiary referral hospital. The outcome was the monthly number of available patient monitors recorded in the equipment management system, which served as the reference value for evaluating five prediction models, including naïve persistence, autoregressive integrated moving average (ARIMA), multivariable linear regression, LSTM, and hybrid LSTM–regression. Model performance was evaluated using root mean squared error (RMSE), mean absolute error (MAE), and mean absolute percentage error (MAPE). Across a 36-month study period, the mean monthly surgical volume was 1960 (SD 182) procedures, with a mean operative duration of 105.0 (SD 3.5) minutes. Weighted multivariable regression showed that service age (standardized *β* = 0.72, 95% CI 0.66–0.78), maintenance frequency (standardized *β* = 0.09, 95% CI 0.03–0.15), and surgical volume (standardized *β* = 0.15, 95% CI 0.09–0.21) were positively associated with available patient monitor, whereas mean operative duration was inversely associated (standardized *β* = −0.19, 95% CI −0.26 to −0.12). The naïve persistence (RMSE 0.17, MAE 0.03, MAPE 0.16%) and ARIMA (RMSE 0.17, MAE 0.04, MAPE 0.21%) showed higher predictive performance, while the multivariable linear regression, LSTM-only, and LSTM–regression models showed relatively higher prediction errors. Beyond predictive accuracy, the model provides an operational framework for transforming routinely collected clinical and equipment data into actionable information for automated resource planning. This study showed that predictive analytics can support hospital automation by enabling proactive patient monitoring and resource planning. Classical statistical models remain robust alternatives for hospital resource forecasting in small-sample settings, while regression-based approaches provide interpretability for operational decision-making.

## 1. Introduction

With the rapid development of smart hospitals and digital health systems, data-driven predictive analytics is increasingly being used to support proactive decision-making, resource optimization, and adaptive planning. Among various applications of predictive hospital automation, healthcare equipment management represents a critical area, as the availability and allocation of medical equipment must be continuously adapted to changing clinical demands. Multiparameter patient monitors continuously measure physiological indicators, supporting the early detection of clinical deterioration and timely intervention across perioperative, intensive care, and general ward settings [1]. Increasing patient acuity, greater surgical complexity, and growing hospital throughput have expanded the need for monitoring beyond traditionally high-dependency environments [2]. However, aligning patient monitor availability with dynamic clinical demand remains a persistent operational challenge. Insufficient monitoring capacity may delay escalation of care, whereas excessive or poorly coordinated deployment may result in underutilization, inefficient capital investment, and increased maintenance burden [3]. Optimizing patient-monitor resource management requires anticipating future equipment availability, considering clinical workload and equipment lifecycle factors [4].

Quantitative approaches to healthcare resource planning have been increasingly explored, including classical statistical models and machine learning methods. A naïve persistence model, which assumes that future demand remains unchanged from the most recent observation, provides a simple benchmark for evaluating more complex forecasting approaches. Traditional time-series approaches such as autoregressive integrated moving average (ARIMA) and exponential smoothing models remain widely used due to their interpretability and robustness in small datasets, while regression-based models can incorporate explanatory variables [5]. Regression-based approaches, such as multiple linear regression, have been used to predict clinical service demand, suggesting their potential to support hospital resource planning and operational decision-making [6]. More recently, machine learning approaches have been increasingly adopted to capture nonlinear temporal dependencies in healthcare systems, including long short-term memory (LSTM) networks and hybrid modeling frameworks [7,8,9,10,11]. However, reported performance varies substantially across study design and sample, and improvements over classical statistical models are not always consistent, particularly in settings with limited observations or weak nonlinear structure.

Existing medical equipment prediction studies focus primarily on clinical demand-side indicators, while equipment-specific determinants are rarely considered, such as utilization intensity, maintenance patterns, and service lifespan [12]. In addition, limited validation in real-world hospital environments restricts their applicability to routine operational decision-making [13,14]. Therefore, integrated analytical frameworks that combine clinical demand and equipment lifecycle attributes are needed to support practical resource planning and intelligent hospital operations.

At Peking University Third Hospital, routinely collected data from clinical information systems and medical equipment management platforms provide a comprehensive record of patient activity and equipment performance. In this study, we conducted a comparative analysis of naïve persistence, classical statistical models, and machine learning approaches, including LSTM and hybrid regression–LSTM, using real-world small-sample hospital operational data. By integrating clinical workload indicators with equipment lifecycle information, this study investigated whether such an approach could provide a practical basis for proactive patient-monitor resource management in data-driven hospital automation.

## 2. Materials and Methods

### 2.1. Study Design and Data Source

We conducted a retrospective, longitudinal modeling study to evaluate predictive approaches for patient monitor availability across 18 operating rooms at Peking University Third Hospital, a high-volume tertiary referral center in northern China. Clinical and equipment-operational data from January 2023 through December 2025 were extracted and integrated from two routinely maintained digital systems: the surgical anesthesia information system and the medical equipment management system. A total of 36 monthly observations, including 70,568 surgical procedures and longitudinal records from 18 patient monitors across 18 operating rooms, were included in the final analyses.

All data were de-identified prior to analysis. Because the study involved secondary analysis of existing administrative data without patient information, institutional review board approval was waived in accordance with institutional policy.

### 2.2. Outcome and Predictors

The primary outcome was the monthly number of patient monitors available in operating rooms, defined as equipment that was functional and not decommissioned or temporarily undergoing maintenance, verification, or repair within each time period. Each operating room was allocated a fixed number of monitors, with one available monitor per room.

Predictors were specified a priori to represent two underlying mechanisms influencing equipment availability, including clinical demand and equipment lifecycle dynamics. Clinical demand was characterized by surgical workload, including monthly surgical volume and mean operative duration, reflecting both the scale and complexity of perioperative activity. Monthly surgical volume was derived by aggregating the number of completed procedures, and mean operative duration (in minutes) was calculated from individual procedure times. Equipment-related predictors captured operational status and aging processes, including mean service age, maintenance frequency, and monthly decommissioning counts. Service age (in years) was calculated as the difference between the production year and the corresponding calendar year and summarized as a monthly average. Maintenance frequency was defined as the number of servicing events per equipment per month, derived from maintenance records, whereas equipment attrition was quantified as the number of monitors removed from service within each month based on equipment status data. A continuous time variable was included to account for temporal progression and potential systematic trends.

### 2.3. Statistical Analysis

A structured comparison was designed to determine whether incorporating clinical and equipment-related predictors or increasing model complexity improved prediction of monthly patient-monitor availability. Five forecasting approaches were evaluated: naïve persistence, autoregressive integrated moving average (ARIMA), multivariable linear regression, LSTM-only, and a hybrid LSTM–regression model. Time-series decomposition using locally estimated scatterplot smoothing (LOESS) was performed as an exploratory analysis to characterize underlying temporal patterns (Method A1–A2). Stationarity of the primary time series was assessed using the Augmented Dickey–Fuller test.

Continuous variables were examined graphically using time-series plots to assess temporal trends and distributional characteristics, and were presented using means and standard deviation (SD) or median and interquartile range (IQR). Statistical analyses were performed using SAS version 9.4 (SAS Institute Inc., Cary, NC, USA) and R version 4.3.2 (R Foundation for Statistical Computing, Vienna, Austria). Two-sided *p*-values less than 0.05 were considered statistically significant.

#### 2.3.1. Prediction Models

##### Naïve Persistence Model

A naïve persistence model was used as a baseline reference for predicting monthly patient monitor availability. This model assumes that the number of patient monitors at time *t +* 1 is equal to the observed value at time *t*. The model was expressed according to Equation (1):

(1) $${\hat{Y}}_{t+1}=Y_{t}$$

##### Autoregressive Integrated Moving Average (ARIMA) Model

An ARIMA model was applied to the monthly time series of patient monitor availability. The model used only historical patient monitor availability records as input and did not include clinical or equipment-related predictors. The expected output was the predicted number of available patient monitors at subsequent time points. The ARIMA model is a univariate time-series approach that captures temporal dependence through autoregressive (AR) terms, differencing (I) for stationarity, and moving average (MA) components. The general ARIMA (*p*, *d*, *q*) model is expressed according to Equation (2):

(2) $$\phi_{p}{\left(B)(1-B\right)}^{d}Y_{t}=\theta_{q}(B)\varepsilon_{t}$$

where *Y_t_* denotes the number of patient monitors at month *t*, *B* is the backshift operator, *p* is the order of the autoregressive component, d is the degree of differencing required to achieve stationarity, and *q* is the order of the moving average component. *ε_t_* represents a white-noise error term.

Model order (*p*, *d*, *q*) was determined based on a combination of visual inspection of autocorrelation and partial autocorrelation functions (ACF and PACF) and the Akaike information criterion (AIC). Stationarity of the time series was assessed using the Augmented Dickey–Fuller (ADF) test, and first-order differencing was applied when necessary. Model parameters, including autoregressive and moving average coefficients as well as the constant term when applicable, were estimated from the training data using maximum likelihood estimation.

##### Multivariable Linear Regression

A multivariable linear regression model was used to examine the associations between surgical workload and equipment operational indicators, and the number of patient monitors. The model was formulated according to Equation (3):

(3) $${Equipment}_{t}=\beta_{0}+\beta_{1}{Surgery}_{t}+\beta_{2}{Duration}_{t}+\beta_{3}{Age}_{t}+\beta_{4}{Maintenance}_{t}+\beta_{5}{Scrapped}_{t}+\epsilon_{t}$$

where *Equipment_t_* denotes the number of patient monitors at month *t*; *Surgery_t_* denotes monthly surgical volume; *Duration_t_* denotes mean operative duration; *Age_t_* denotes mean equipment service age; *Maintenance_t_* denotes maintenance frequency; *Scrapped_t_* denotes the number of equipment removed from service. Results from the regression model are presented as *β* coefficients with 95% confidence intervals (*CIs*). Regression diagnostics included assessment of multicollinearity using variance inflation factors, evaluation of residual normality using the Shapiro–Wilk test, and testing for heteroskedasticity using the Breusch–Pagan test.

If heteroscedasticity was detected in the residuals of the multivariable linear regression model, a Weighted Least Squares (WLS) regression was conducted. The WLS model estimates regression coefficients by assigning weights to each observation, reducing the influence of observations with higher variance and stabilizing residual variance. Weights were calculated as the inverse of the squared residuals from the initial multivariable linear regression according to Equation (4):

(4) $$w_{i}=\frac{1}{{\hat{\sigma }}_{i}^{2}}=\frac{1}{{\left(Y_{i}-{\hat{Y}}_{i}\right)}^{2}}$$

where *Y_i_* is the observed value; ${\hat{Y}}_{i}$ is the predicted value from the linear regression; and represents the variance of the residual for observation *i*.

##### LSTM-Regression Prediction Model

**Figure A1:**
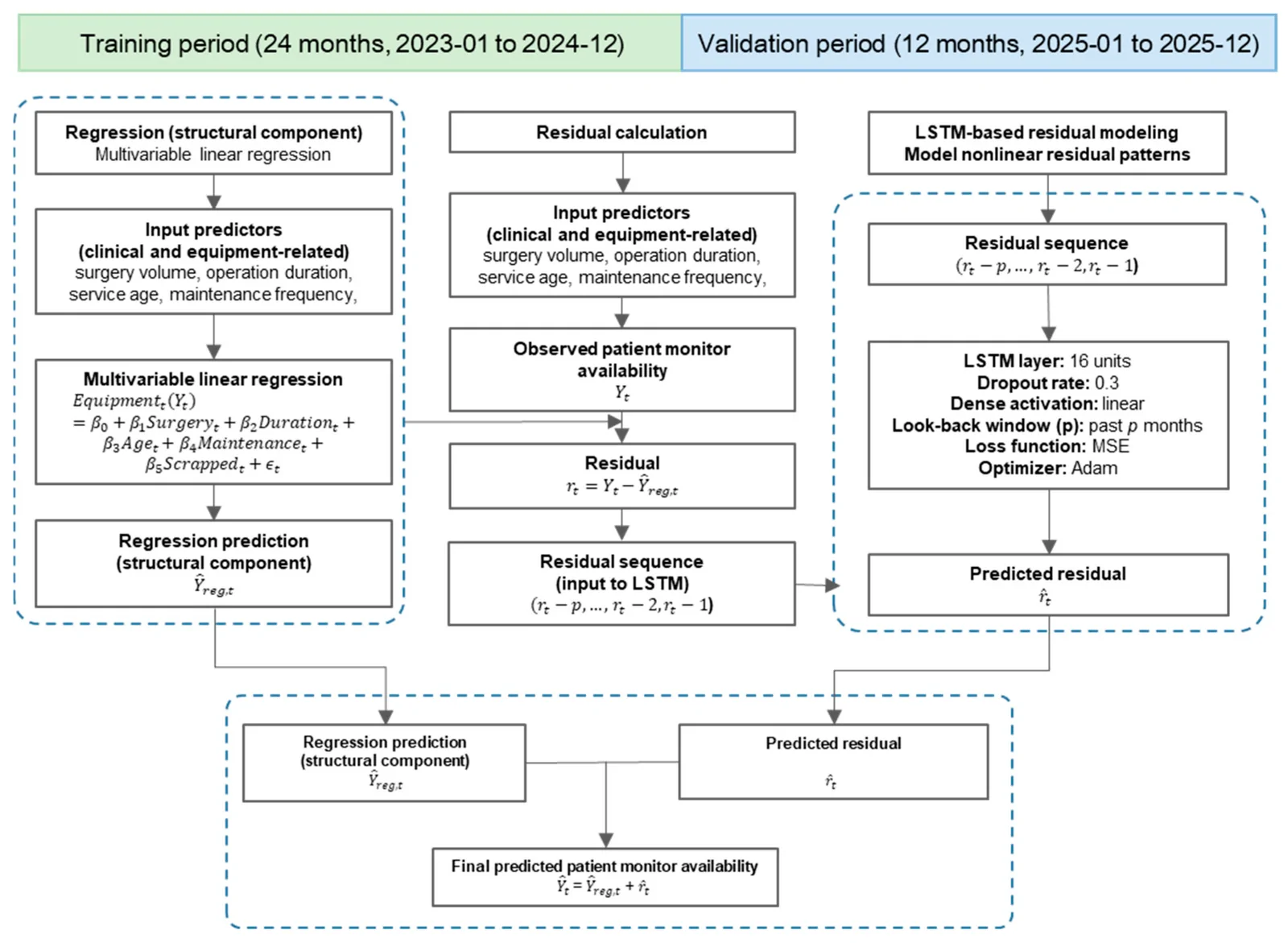
Framework of the hybrid regression-LSTM model for patient monitor availability forecasting.

The hybrid LSTM–regression model used a residual learning framework, where multivariable linear regression modeled the structural component, and LSTM modeled the remaining nonlinear residual component. The regression model first generated predictions based on clinical and equipment-related predictors. The differences between observed values and regression-based predictions were calculated as residuals, which were normalized using a min–max approach [15,16] described in Method A3, and subsequently modeled using the LSTM network (Figure A1) to capture nonlinear temporal dependencies. The predicted residuals were transformed back to the original scale and then combined with the regression-based structural prediction to obtain the final predicted value according to Equation (5).

(5) $${\hat{Y}}_{t}={\hat{Y}}_{regression,t}+{\hat{e}}_{LSTM,t}$$

where ${\hat{Y}}_{regression,t}$ denotes the predicted value from the multivariable linear regression model and ${\hat{e}}_{LSTM,t}$ denotes the predicted nonlinear residual after inverse transformation to the original scale. This combined approach preserves structural interpretability while incorporating nonlinear corrections.

The LSTM network consisted of a single LSTM hidden layer with 16 LSTM units, a dropout layer with a rate of 0.3 to reduce overfitting, and a fully connected dense layer with linear activation to generate continuous residual predictions, totaling 321 trainable parameters. The input sequence length was set to one time step because of the limited number of monthly observations. The model was trained using the Adam optimizer with a learning rate of 0.001, a batch size of 4, and a maximum of 200 epochs. Early stopping was applied based on validation loss with a patience of 10 epochs to prevent overfitting. This relatively compact architecture was selected to balance model flexibility and parsimony given the limited time-series length. The weight update rule was defined according to Equation (6):

(6) $$w\leftarrow w-\alpha \frac{v_{dw}}{\sqrt{s_{dw}}+\epsilon }$$

where *α* represents the learning rate (0.001); *v_dw_* and *s_dw_* represent exponentially weighted moving averages of gradients and squared gradients, respectively; *ϵ* is a small constant to prevent numerical instability. Early stopping based on validation loss was implemented to reduce overfitting.

#### 2.3.2. Model Training and Validation

Data from January 2023 through December 2024 were used for model training, and data from January 2025 through December 2025 were reserved for temporal validation.

#### 2.3.3. Model Performance Evaluation

Model performance was assessed using root mean squared error (RMSE), mean absolute error (MAE), and mean absolute percentage error (MAPE). The Diebold–Mariano test was used to compare predictive accuracy between models.

## 3. Results

### 3.1. Baseline Characteristics

Between January 2023 and December 2025, a total of 36 monthly observations were analyzed across 18 operating rooms. The mean monthly surgical volume was 1960.2 (SD 182.6) procedures, with an average operative duration of 105.0 (SD 3.5) minutes. Mean service age increased from 3.4 (SD 0.5) years during the 2023–2024 training period to 4.8 (SD 0.3) years in the 2025 validation period. Monthly maintenance events had a median of 1 (IQR 0–2), and no equipment was decommissioned throughout the study. Mean monthly patient monitors increased from 17.4 (SD 0.5) units during training to 18.0 units during validation (Table 1).

### 3.2. Temporal Patterns and Model Assumptions

**Figure 1:**
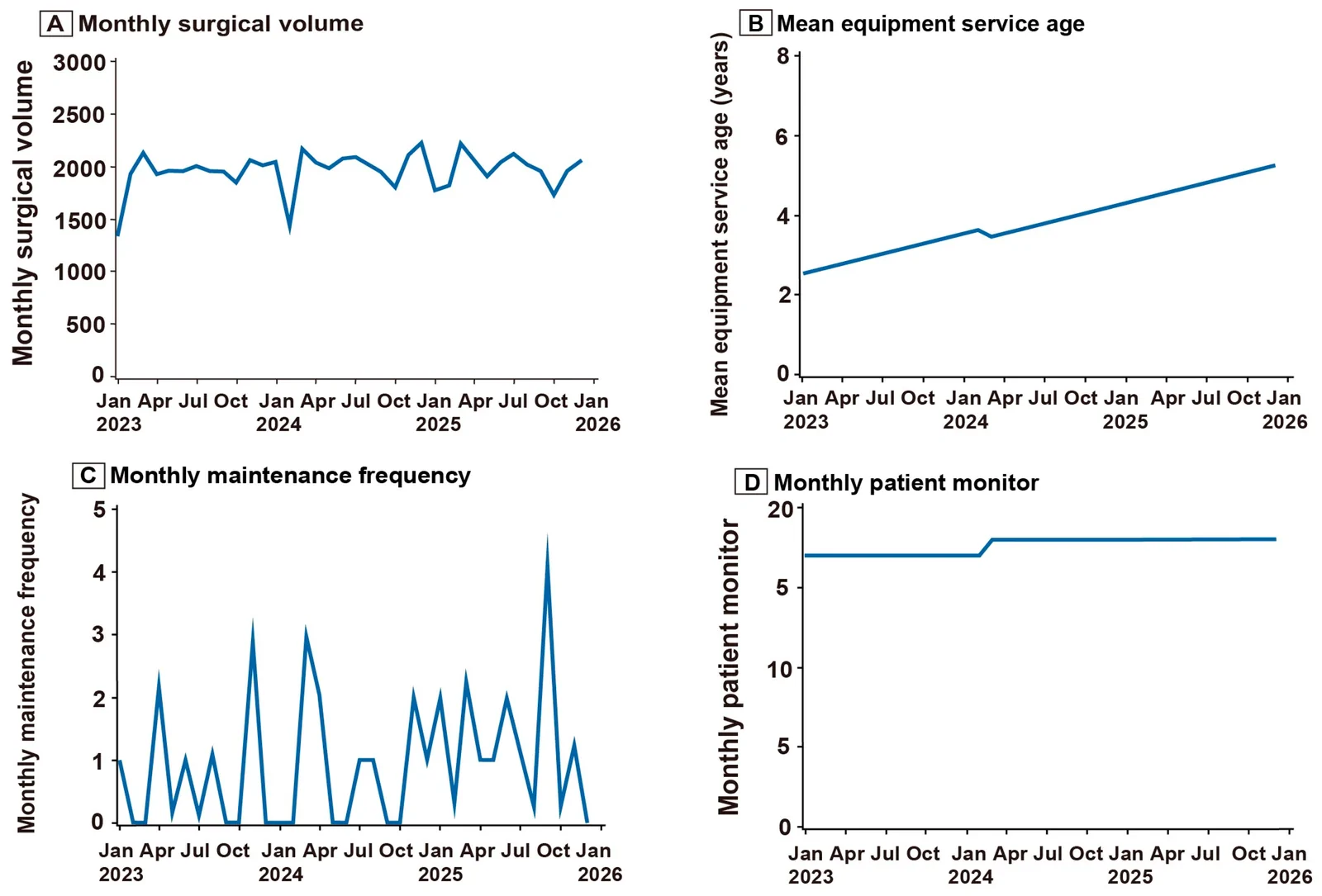
Temporal trends for surgical workload, equipment operation, and available patient monitors. Panels (**A**–**D**) show monthly trends in surgical volume, mean equipment service age, maintenance frequency, and patient monitors.

**Figure A2:**
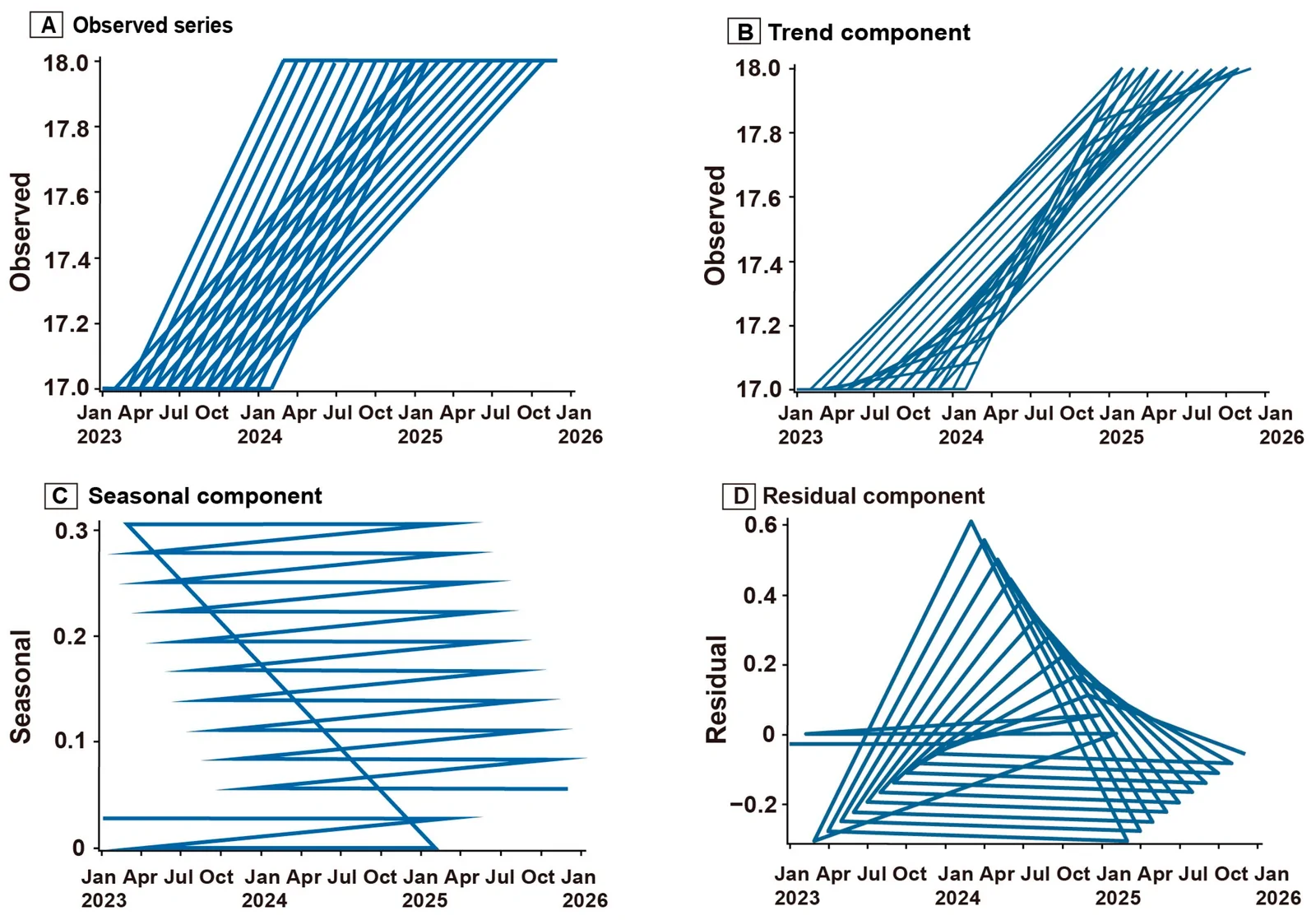
Seasonal–trend decomposition for surgical workload, equipment operation, and available patient monitor. Panels (**A**–**D**) present the seasonal–trend decomposition of patient monitor availability using LOESS decomposition. The decomposition separates the original time series into four components: the observed series represents the actual monthly number of available patient monitors; the trend component reflects the long-term direction of change; the seasonal component represents periodic fluctuations over time; and the residual component indicates short-term variations unexplained by trend and seasonality. The decomposition allows visual assessment of underlying temporal patterns before model development.

Monthly surgical volume and maintenance frequency fluctuated without a consistent long-term trend, and mean equipment service age and patient monitors increased over time (Figure 1A–D). Seasonal–trend decomposition using locally estimated scatterplot smoothing separated the patient monitor series into additive trend, seasonal, and residual components, indicating a gradual upward trend, minor seasonal fluctuations, and residual variability centered around zero (Figure A2). The Augmented Dickey–Fuller test showed non-stationarity of the monthly patient monitor series (all *p* > 0.05, Table A1).

**Figure A4:**
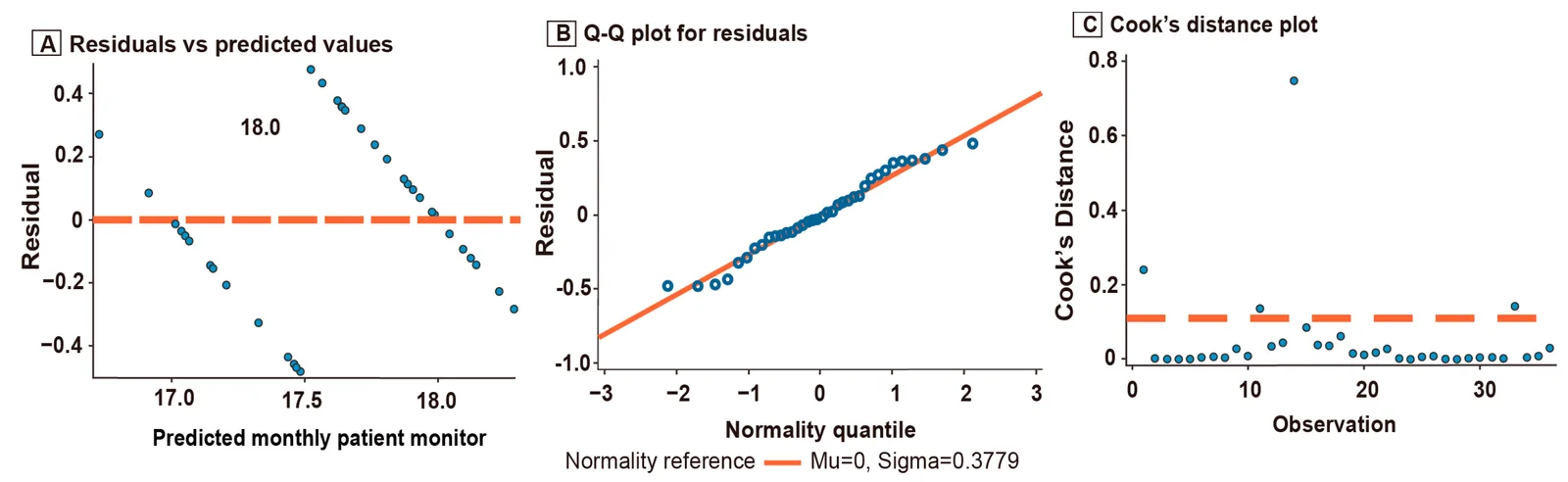
Diagnostic evaluations of the multivariable linear regression model. Panels (**A**–**C**) show residuals versus predicted values, a QQ plot of residuals, and Cook’s distance for influential observations.

Collinearity diagnostics showed tolerance values ranging from 0.76 to 0.92 and variance inflation factors between 1.09 and 1.31 (Table A3), with no substantial multicollinearity among indicators. Pearson correlation analysis showed a strong positive association between mean equipment service age and patient monitors (r = 0.89), and other correlations were smaller in magnitude (Figure A2). Diagnostic assessment of the initial ordinary least squares model showed residuals centered around zero, with some variation in spread across fitted values suggesting mild heteroscedasticity. The Q–Q plot showed approximate normality of residuals, and Cook’s distance identified a few potentially influential observations with no substantial change in coefficient stability (Figure A4; Table A3 and Table A4).

### 3.3. Multivariable Linear Regression Analysis

Weighted least squares multivariable linear regression showed that mean equipment service age was independently associated with monthly patient monitors (standardized *β* 0.72, 95% CI 0.66 to 0.78) (Table 2). Monthly maintenance frequency was also associated with increased patient monitors (standardized *β* 0.09, 95% CI 0.03 to 0.15). Mean operative duration was associated with decreased patient monitors (standardized *β* −0.19, 95% CI −0.26 to −0.12). Monthly surgical volume was also associated with increased patient monitors (standardized *β* 0.15, 95% CI 0.09 to 0.21). Variance inflation factors ranged from 1.06 to 3.05 in the final model, indicating the absence of substantial multicollinearity.

### 3.4. Predictive Performance for Prediction Models

The naive persistence model provided a simple reference and yielded an RMSE of 0.17, MAE of 0.03, and MAPE of 0.16% (Table 3). The ARIMA model showed similar performance, with an RMSE of 0.17, MAE of 0.04, and MAPE of 0.21%. The linear regression and LSTM-only models showed higher prediction errors compared with the naïve and ARIMA models (linear regression: RMSE 0.27, MAE 0.21, MAPE 1.21%; LSTM-only: RMSE 0.280, MAE 0.215, MAPE 1.252%), whereas the hybrid model, combining regression-based structural predictions with LSTM-modeled residuals showed reductions in RMSE and MAE compared with linear regression or LSTM-only models (RMSE 0.19; MAE 0.14; MAPE 0.78%). The Diebold–Mariano test showed a reduction in absolute prediction error for the hybrid model compared with linear regression (mean difference 0.089, *t* = 7.49, *p* < 0.001), the LSTM-only model (mean difference 0.339, *t* = 8.89, *p* < 0.001), while no statistically significant difference was observed between the hybrid and naïve persistence model (mean difference 0.009, *t* = 0.29, *p* = 0.77) and ARIMA model (mean difference 0.006, *t* = 0.04, *p* = 0.74).

**Figure 2:**
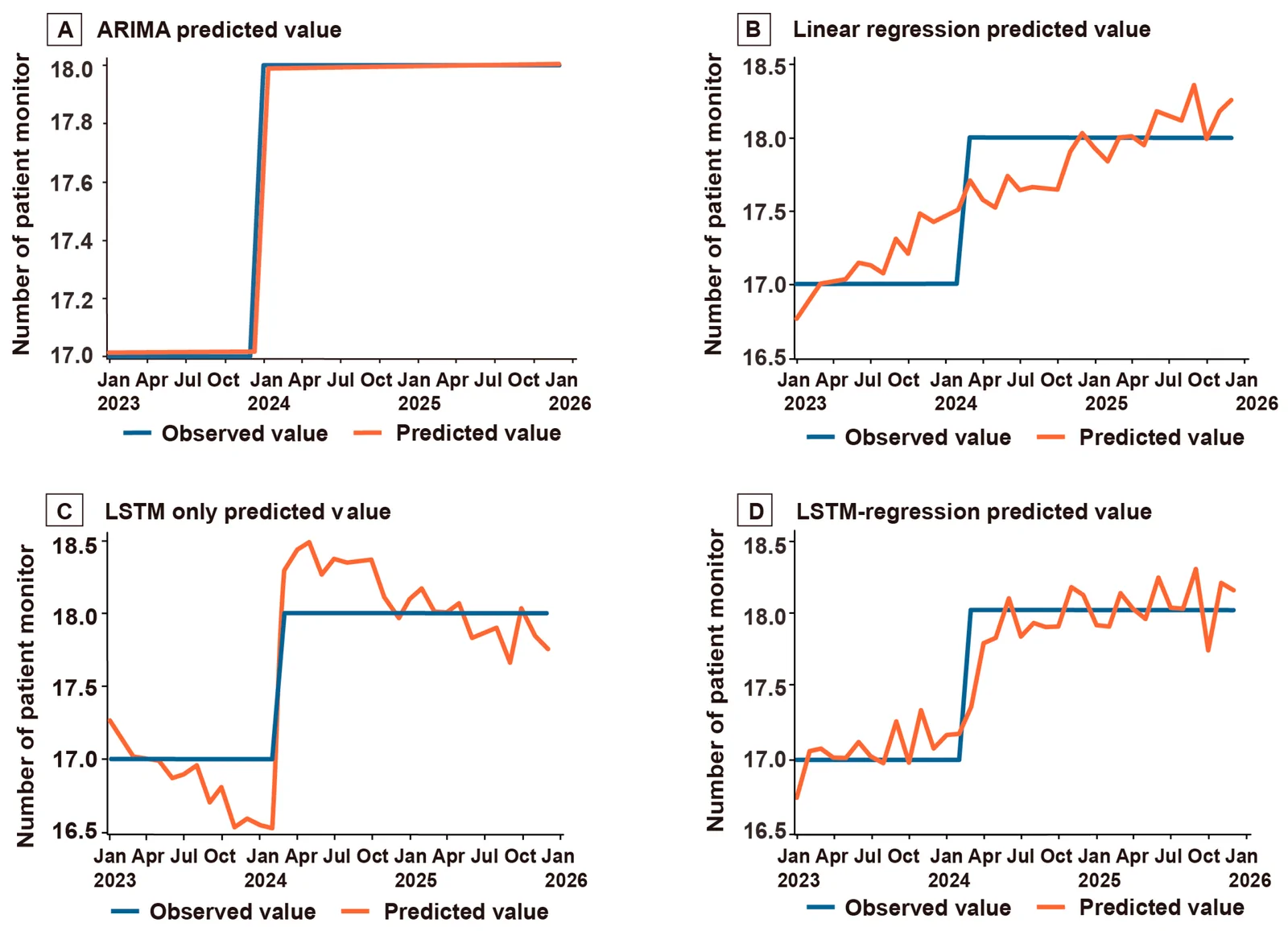
Performance of the prediction model for available patient monitor. Panels (**A**–**D**) show predicted patient monitor availability from the ARIMA model, multivariable linear regression model, LSTM model, and hybrid regression–LSTM model, respectively.

The observed and predicted patient monitor availability generated by different models were shown in Figure 2. The ARIMA model showed relatively stable predictions and a similar trend to the observed change in monitor availability. The multivariable linear regression model reflected the overall increasing trend but showed larger deviations from the observed values. The LSTM-only model showed greater fluctuations around the observed values. The hybrid regression–LSTM model showed predictions closer to the observed values than the linear regression and LSTM-only models by combining regression-based structural estimation with nonlinear residual modeling.

Model performance metrics for the naïve persistence model, ARIMA model, linear regression, LSTM, and hybrid models. RMSE, root mean square error; MAE, mean absolute error; MAPE, mean absolute percentage error.

## 4. Discussion

This study evaluated the role of predictive analytics in supporting hospital automation through operating room equipment resource management. By comparing multiple forecasting approaches, including multivariable linear regression, ARIMA model, and machine learning-based methods, we investigated their applicability for predicting patient monitor availability in a tertiary referral hospital. Although the hybrid LSTM–regression model introduces additional modeling complexity by combining structural and residual components, its predictive performance was not superior to simpler benchmark models, and ARIMA achieved accuracy comparable to the naïve persistence approach. Equipment service age was the dominant factor associated with patient monitor availability, with surgical volume, maintenance frequency, and operative duration serving as independent predictors. These findings suggest that with constrained sample sizes and relatively stable operational patterns, robust and interpretable forecasting approaches may be more valuable than increased model complexity for supporting proactive resource planning.

### 4.1. Previous Study Review

Predictive analytics has been increasingly used in healthcare operational management, particularly for resource planning, equipment allocation, and logistics optimization. Previous studies have shown that forecasting approaches can support decision-making across various healthcare scenarios. During the COVID-19 pandemic, deep learning models such as multilayer LSTM networks showed potential for predicting respiratory equipment demand by capturing complex temporal patterns under rapidly changing conditions [11]. A hybrid prediction model combining bidirectional LSTM and genetic algorithm-supported support vector regression has been applied to forecast intensive care resource requirements, including medical equipment and staffing needs, providing support for timely allocation during public health emergencies [10]. Beyond emergency scenarios, forecasting methods have also been explored for routine medical supply management, with approaches such as fuzzy time series models demonstrating effectiveness in predicting inventory requirements and improving supply planning [9]. However, the optimal forecasting strategy depends strongly on data characteristics and operational context. Classical time-series models such as ARIMA remain widely used because of their interpretability and robustness, particularly when historical observations are limited and temporal patterns are relatively stable [17,18]. ARIMA models often perform competitively in short historical series and may be difficult to outperform when external covariates are limited and temporal structure is stable [17]. These findings suggest that in healthcare operational data with limited observations and relatively smooth temporal evolution, classical statistical models may have comparable or superior performance to more complex machine learning methods.

### 4.2. Structural and Operational Determinants of Patient Monitors

Equipment service age showed a stronger association with the number of patient monitors, suggesting that equipment lifecycle characteristics represent important operational factors for understanding hospital equipment capacity. Over the study period, the hospital gradually acquired additional monitors, and the average service age of monitors increased concurrently. Several studies on healthcare technology management have highlighted lifecycle-informed frameworks for optimizing equipment utilization and long-term capital planning [19,20,21,22]. A recent systematic review reported that equipment service life is associated with observed performance, efficiency, and uptime, providing a basis for maintenance scheduling, replacement planning, and allocation strategies in hospital settings [20].

Maintenance frequency was positively associated with the number of available patient monitors. This finding suggests that higher maintenance frequency may reflect both increased failures and greater utilization intensity [23], potentially influencing decisions regarding equipment redundancy, maintenance scheduling, and future capacity planning. Frequent maintenance can indicate recurrent performance issues requiring backup capacity, while also capturing the effects of intensive use that accelerates wear and increases servicing needs. Evidence from recent studies indicates that maintenance demand and failure frequency are closely linked to equipment reliability and availability, with implications for service readiness and healthcare delivery [20,22]. Maintenance frequency functions as a composite indicator of both failure burden and utilization pressure, thereby influencing equipment redundancy requirements and ultimately shaping configuration decisions in clinical settings.

Mean operative duration showed an inverse association with the number of patient monitors. A possible explanation is that longer procedures tend to be concentrated in a smaller number of operating rooms, whereas shorter procedures allow higher parallel use of multiple rooms, resulting in greater monitor allocation [24,25], which necessitates a larger number of operating rooms and corresponding monitor allocation. Given the limited dataset of 36 monthly aggregated observations, these explanations remain descriptive, and the precise operational mechanisms cannot be determined.

Monthly surgical volume was positively associated with available patient monitor availability, indicating that increasing surgical demand requires expanded monitoring capacity to support concurrent procedures and maintain perioperative safety. Under a one-monitor-per-operating-room configuration, higher surgical volume might be accompanied by increased operating room utilization and parallel scheduling, leading to a greater number of monitors under a one-monitor-per-operating-room configuration [26]. Moreover, higher surgical volume is associated with increased equipment utilization, which may accelerate wear and elevate the risk of equipment failure and maintenance demand [20,27], thereby necessitating additional equipment to ensure service continuity and avoid operational disruptions. These findings suggest that surgical volume contributes to the need for predictive resource planning in operating room environments.

### 4.3. Comparison of Prediction Models

The comparison of forecasting approaches in this study highlights the importance of selecting models according to operational context rather than relying solely on algorithmic complexity. The naïve persistence and ARIMA models showed the best predictive performance, suggesting that temporal continuity and stable historical patterns provided substantial information for forecasting patient monitor availability in this setting. Although the hybrid regression–LSTM model showed comparable performance to the naïve persistence model under the current stable operational conditions, it provides a framework for integrating operational predictors with temporal residual modeling, which may be valuable in more variable settings. These findings are consistent with previous evidence that classical time-series models remain effective when operational processes exhibit relatively stable temporal structures and limited external variability [17,18].

Multivariable linear regression provides interpretable estimates of associations between equipment demand and operational drivers. Linear models are limited in capturing nonlinear temporal dependencies and unobserved dynamic effects, which may reduce predictive flexibility in time-evolving systems [28]. Although regression-based approaches may not always achieve the lowest prediction error, their interpretability provides important advantages for hospital resource management, where understanding contributing factors is essential for planning, maintenance strategies, and equipment lifecycle decisions.

LSTM-based models are designed to capture nonlinear temporal dependencies and long-term sequence patterns, showing promising performance in complex and high-frequency healthcare forecasting scenarios. However, their advantages are strongly influenced by data scale, temporal complexity, and the presence of nonlinear patterns. In the current study, the limited number of monthly observations and relatively stable operational processes may have constrained the ability of LSTM-based approaches to provide additional predictive benefit over simpler models [29].

These findings have practical implications for predictive hospital automation and equipment management. In real-world healthcare environments, forecasting approaches should balance predictive accuracy, robustness, interpretability, and implementation feasibility. For small-sample operational datasets, simpler statistical models may provide reliable decision-support information for proactive equipment planning, while more complex machine learning approaches may become advantageous when larger datasets and more dynamic operational conditions are available. Therefore, predictive analytics should be viewed as an enabling tool for hospital resource management rather than a requirement for adopting increasingly complex algorithms.

### 4.4. Limitations

This study has several limitations. First, it was conducted in a single tertiary hospital, limiting generalizability to institutions with different capacity, workflow, or administrative structures. Second, the observation period spanned 36 months, which may not fully capture longer-term life-cycle dynamics of patient monitors or rare operational disruptions. In addition, the dataset included only 24 months for training and 12 months for validation, which limits the ability of LSTM networks to reliably capture complex temporal dependencies. The hybrid regression–LSTM framework primarily served to model residual fluctuations and should be interpreted as exploratory. Moreover, the number of available patient monitors showed limited variation during the study period (17–18 monitors), which may have reduced the forecasting complexity and contributed to the comparable performance of simpler models. These findings highlight that, under small-sample conditions, the LSTM component should be interpreted with caution, and generalization of the results may be limited. Third, patient-level outcomes, such as perioperative complications, and economic information, such as acquisition and maintenance costs, were not available and were not incorporated into model development, limiting assessment of how these factors influence monitor allocation and model predictive performance. Fourth, although the hybrid model improves predictive performance, the small integer range of monitor counts may limit its ability to distinguish minor differences in operational allocation decisions. Future studies could explore integrating patient-level utilization data and multi-center datasets to enhance model generalizability and support more granular equipment allocation decisions. Finally, no monitor decommissioning events occurred during the study period, limiting the assessment of available reductions due to equipment failure. Future studies should investigate the impact of patient-level clinical outcomes and economic considerations on monitor allocation, incorporating these variables to evaluate their contribution to model performance and to support cost-effective, clinically informed equipment management.

## 5. Conclusions

We evaluated predictive approaches for supporting operating room equipment management by comparing prediction models for patient monitor availability in a tertiary referral hospital. The comparative analysis showed that model performance varied across approaches, and the proposed hybrid regression–LSTM model did not show substantially better performance than simpler baseline models under the limited and relatively stable longitudinal dataset. Equipment service age was the strongest predictor, with maintenance frequency, operative duration, and surgical volume also contributing to variations in patient monitor availability. These findings suggest that simpler forecasting models might have competitive performance under small-sample and relatively stable operational conditions, indicating that predictive analytics can provide practical decision support for proactive equipment planning and hospital resource management.

## Figures and Tables

**Table 1:** Baseline characteristics of surgical workload and equipment operational indicators (Overall, Training, and Validation datasets; N = 36 months).

Predictor	Overall (2023–2025, N = 36 Months)	Training (2023–2024, N = 24 Months)	Validation (2025, N = 12 Months)
Monthly surgical volume	1960.2 ± 181.6	1957.3 ± 200.1	1966.2 ± 145.5
Mean operative duration (min)	105.0 ± 3.5	105.5 ± 4.0	104.0 ± 2.3
Mean equipment service age (years)	3.9 ± 0.8	3.4 ± 0.5	4.8 ± 0.3
Monthly maintenance frequency	1 (0–1.5)	0 (0–1)	1 (0–2)
Monthly scrapped equipment	0	0	0
Monthly patient monitor	17.6 ± 0.5	17.4 ± 0.5	18 ± 0

**Table 2:** Multivariable linear regression for monthly patient monitor.

Predictor	*β* (95% CI)	Standardized *β* (95% CI)	VIF
Monthly surgical volume	0 (0, 0)	0.15 (0.09, 0.21)	1.06
Mean operative duration (min)	−0.03 (−0.04, −0.02)	−0.19 (−0.26, −0.12)	3.05
Mean equipment service age (years)	0.45 (0.42, 0.49)	0.72 (0.66, 0.78)	2.79
Monthly maintenance frequency	0.04 (0.02, 0.07)	0.09 (0.03, 0.15)	1.21

**Table 3:** Predictive performance comparison.

Model	RMSE	MAE	MAPE (%)
Naive persistence model	0.169	0.029	0.159
ARIMA model	0.166	0.036	0.209
Linear regression model	0.265	0.213	1.210
LSTM only	0.280	0.215	1.252
Hybrid model	0.185	0.138	0.782

**Figure A3:**
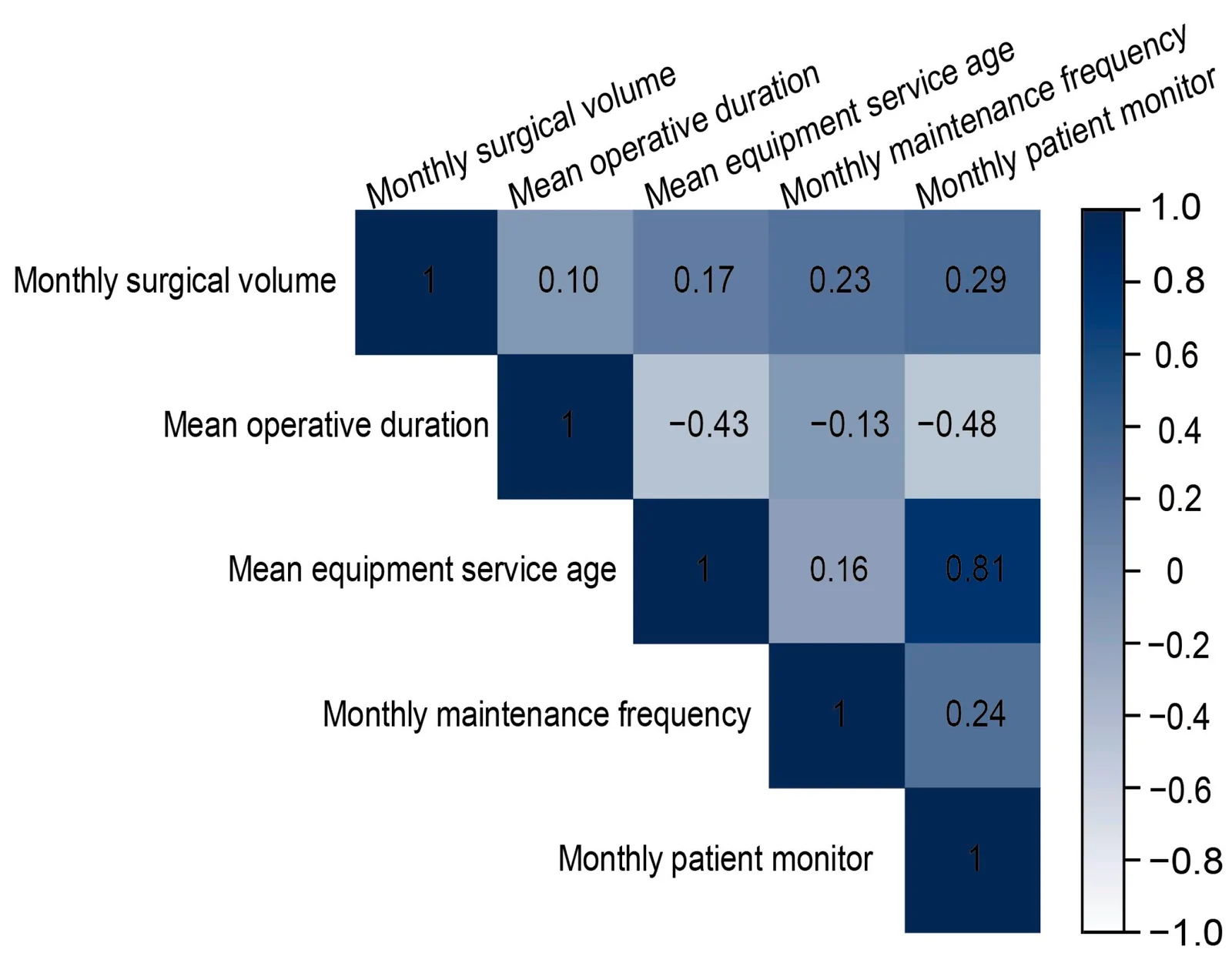
Correlation matrix among surgical workload and equipment operational indicators.

## Data Availability

The raw data supporting the conclusions of this article will be made available by the authors on request.

## Abbreviations

The following abbreviations are used in this manuscript:

SD	Standard Deviation
IQR	Interquartile Range
LSTM	Long Short-Term Memory
95% CI	95% Confidence Intervals
RMSE	Root Mean Squared Error
MAE	Mean Absolute Error
MAPE	Mean Absolute Percentage Error

## Appendix A

**Method A1. Time-series decomposition**

Seasonal–trend decomposition using locally estimated scatterplot smoothing was performed to decompose the monthly patient monitor time series into additive trend, seasonal, and residual components. The trend component was used to characterize long-term structural changes, whereas the residual component was used to capture short-term deviations not explained by seasonal or trend patterns.

**Method A2. Hybrid modeling strategy**

The modeling strategy was informed by the premise that patient monitor demand may reflect both surgical workload and equipment operational characteristics. Factors such as equipment age, decommissioning patterns, and maintenance frequency were considered to potentially influence replacement and allocation needs. Accordingly, workload-related and equipment-related variables were incorporated into model development.

To account for structural associations and potential nonlinear temporal patterns, a hybrid modeling approach was used that combined multivariable linear regression with nonlinear time-series modeling based on a long short-term memory (LSTM) network.

**Method A3. Residual generated from multivariate linear regression**

To improve numerical stability and facilitate LSTM optimization, the residual input sequence was normalized using min–max scaling to a range of 0 to 1 [18,19] according to Equation:

$$X_{norm}=\frac{X-X_{min}}{X_{max}-X_{min}}$$

where *X_min_* and *X_max_* denote the minimum and maximum values calculated from the training dataset. Normalization parameters were derived exclusively from the training dataset and subsequently applied to the validation dataset to prevent information leakage.

**Table A1:** Augmented Dickey–Fuller test for time-series stationarity of monthly patient monitor.

Series	Lag	Rho	Pr < Rho	Tau	P < Tau	F	Pr > F	Stationarity
Observed_count	0	0.0548	0.6889	0.96	0.9077	–	–	No
Observed_count	1	0.0548	0.6887	0.96	0.9075	–	–	No
Observed_count (single mean)	0	−2.5	0.7055	−1.23	0.6485	1.27	0.7514	No
Observed_count (single mean)	1	−2.6154	0.6903	−1.25	0.6404	1.29	0.7459	No
Observed_count (trend)	0	−6.4912	0.6728	−1.71	0.727	1.54	0.8682	No
Observed_count (trend)	1	−7.3791	0.5911	−1.68	0.7394	1.52	0.872	No

Lag, lagged term; Rho, autoregressive coefficient; Tau, test statistic for unit root; F = F-statistic for joint significance of coefficients. Stationarity indicates whether the null hypothesis of a unit root is rejected (No = non-stationary).

**Table A2:** Collinearity diagnostics.

Predictor	Tolerance	Variance Inflation Factor (VIF)
Monthly surgical volume	0.886	1.129
Mean operative duration (min)	0.770	1.299
Mean equipment service age (years)	0.761	1.314
Monthly maintenance frequency	0.920	1.087

Tolerance = 1 − R^2^ when each predictor is regressed on the remaining predictors.

**Table A3:** Breusch–Pagan test for heteroscedasticity.

Test	Chi-Square	*p* Value
Breusch–Pagan	10.75	0.041

The Breusch–Pagan test evaluates the null hypothesis of homoskedasticity. A *p*-value < 0.05 indicates heteroskedasticity.

**Table A4:** Shapiro–Wilk test for residual normality.

Test	W Statistic	*p* Value
Shapiro–Wilk	0.968	0.384

The Shapiro–Wilk test evaluates the null hypothesis that residuals follow a normal distribution. A *p*-value < 0.05 indicates deviation from normality.
